# Impact of Excision Type, Cone Volume, and Dimensions on Persistence/Recurrence of Cervical Intraepithelial Neoplasia 2–3

**DOI:** 10.3390/life14080968

**Published:** 2024-07-31

**Authors:** Gonzalo Arturo Medina Bueno, Maria Eulalia Fernández-Montolí, Fatima Heydari, Jordi Ponce, Sara Tous, Judith Peñafiel

**Affiliations:** 1 Medicine and Translational Research Doctorate Program, Faculty of Medicine and Health Sciences, University of Barcelona, 08036 Barcelona, Spain; fatimaheydari.bcn@gmail.com; 2 Gynecology Department, Hospital Universitari de Bellvitge, IDIBELL, Universitat de Barcelona, L’Hospitalet de Llobregat, 08907 Barcelona, Spain; jponce@bellvitgehospital.cat; 3 Cancer Epidemiology Research Program, Catalan Institute of Oncology (ICO), Bellvitge Biomedical Research Institute (IDIBELL), L’Hospitalet de Llobregat, 08908 Barcelona, Spain; stous@iconcologia.net (S.T.); jpenafiel@idibell.cat (J.P.)

**Keywords:** cervical intraepithelial neoplasia, HPV, margin status, recurrence, uterine cervical dysplasia

## Abstract

The objective of this study was to evaluate the relationship between the excision type and the persistence/recurrence of CIN2–3. A total of 227 women with CIN2–3 who were treated with LLETZ were evaluated. The types of excision according to the IFCPC 2011, volume, cone dimensions, margins of resection, post-cone high-risk human papillomavirus (HR-HPV) status, and viral load were studied. The time to recurrence was assessed using Kaplan–Meier curves. Persistent/recurrent CIN2–3 was found in 12 cases (5.2%). Type 1 excision was performed in 107 patients, with 7 recurrences (6.5%); type 2 excision in 74 patients, with 4 recurrences (5.4%); and type 3 excision in 46 patients, with 1 recurrence (2.1%). The percentage of clear margins in type 1 excisions was 44.9%, that in type 2 excisions was 59.5%, and that in type 3 excisions was 69.6% (*p* = 0.008). Type 1 excision was associated with 28.5% post-LLETZ HR-HPV positivity, that in type 2 reached 20.6%, and that in type 3 reached 11.4%; this difference was non-significant (*p* = 0.24). (4) Conclusions: Type 3 excision was associated with a larger proportion of clear margins and lower post-cone HR-HPV positivity, with a lower incidence of the persistence/recurrence of CIN2–3.

## 1. Introduction

Women with high-grade cervical intraepithelial neoplasia (CIN2–3) should be treated to prevent its progression to cancer [1]; however, there are also circumstances whereby observation is appropriate. Different treatment modalities are used for the transformation zone, including ablative techniques (cryotherapy, laser ablation, or thermal ablation) and excision techniques (large-loop excision of the transformation zone, LLETZ, or conization with a cold knife). Medical management is an alternative to surgery/observation, and imiquimod and 5-fluorouracil cream are considered as acceptable treatment methods [2]. The choice of treatment depends on the type of transformation zone, the size and location of the lesion, and other crucial factors, including factors related to the patient, treating physician, and procedure [3]. LLETZ is intended to remove the entire transformation zone, including the entire lesion, such that it can be evaluated histologically [4]. Its advantage is that it allows for histological confirmation and assessment of the surgical margins and allows stromal invasion to be ruled out [5]. Its drawbacks relate to the difficulty in performing a histological assessment of the margins due to thermal damage [5]. According to the 2011 guidelines of the International Federation of Cervical Pathology and Colposcopy (IFCPC), the transformation zone (TZ) is classified as a type 1 TZ when it is located in the exocervix and is completely visible. A type 2 TZ is defined as one that is located in the exocervix but is partially introduced into the endocervical canal and is visible in its entirety. A type 3 TZ is one that is located in the endocervical canal and not completely visible [6]. The most appropriate type of excision can be chosen according to the type of transformation zone, with the aim of achieving complete excision of the lesion. According to the modified IFCPC classification, three types of excision are possible: if the resection of a type 1 transformation zone is performed, the diathermic loop should not include the endocervical canal or exceed 8 mm in depth; if the resection is from a type 2 transformation zone, 10 to 15 mm of the endocervical canal is resected; and, when the resection is from a type 3 transformation zone, the endocervical canal is extracted to a depth of 15 to 25 mm [4]. There is controversy regarding the appropriate depth and dimensions of the conization specimen, and few studies have compared the types of excision with regard to the possibility of recurrence. The objective of the present study was to evaluate the relationship between the type of conization according to the IFCPC and the volume and dimensions of the LLETZ (circumference, thickness, and length), in addition to the persistence/recurrence of CIN2–3, the presence of involved margins in conization specimens collected using a diathermic loop, and post-LLETZ HR-HPV positivity.

## 2. Materials and Methods

**Figure 1:**
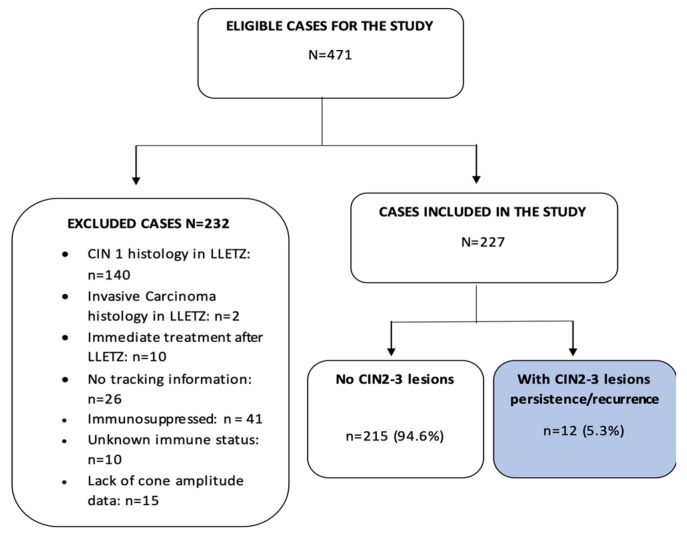
Process for the inclusion and exclusion of patients from the present study. CIN = cervical intraepithelial neoplasm, LLETZ = large-loop excision of the transformation zone.

This study included conization samples from patients with a histological diagnosis of CIN2–3 who were treated in the Cervical Pathology Unit of the Gynecology Department of Bellvitge University Hospital, Hospitalet de Llobregat, Barcelona. The patient inclusion period was January 1996 to December 2016. Women who had at least one follow-up control after LLETZ were included. The study excluded patients with less than one follow-up control after LLETZ (n = 10), as well as patients with immunosuppression (n = 41), unknown immune status (n = 10), and missing second excision data (n = 2). Finally, of the 471 eligible cases, 227 patients (51.4%) were included. The inclusion/exclusion procedure is shown in Figure 1. The patient data were appropriately anonymized and protected in accordance with national regulations. This study was approved by the Clinical Research Ethics Committee of the Bellvitge University Hospital (dated 14 February 2019).

### 2.1. Surgical Procedure

Conization was performed using a diathermic loop after applying local paracervical anesthesia and Lugol to the surface of the cervix. The diameter of the loop was chosen according to the colposcopic appearance of the lesion to be removed. Type 1 excision was performed when a type 1 transformation zone was found; type 2 excision was performed if the transformation zone was of type 2; and type 3 excision was performed if the transformation zone was of type 3. In women over 35 years of age or in whom a squamocolumnar junction was not visible, a second pass of the endocervical canal (top hat) was performed with a 5 mm loop. Hemostatic coagulation was performed with a spherical electrode. The samples were oriented with a point at the 12 o’clock position.

### 2.2. Patient Testing Methodology and Follow-Up

Ectocervical and endocervical smears were obtained using the conventional Papanicolaou staining method. The cytological findings were evaluated according to the 1989 or 2001 Bethesda System terminology [7].

Colposcopy was performed using a binocular Carl Zeiss colposcope (Jena, Germany). Acetic acid (5%) was initially applied to the cervix with a cotton swab, followed by Lugol. Women with abnormal cytology (ASC-US or higher) or a positive HR-HPV test result underwent colposcopy and a biopsy. An endocervical curettage was performed if the transformation zone was of type 3.

The samples for the HPV test were processed using the Digene test (Gaithersburg, MD, USA), which involves signal-amplified hybridization capture through the chemiluminescent detection of an HR-HPV type-specific RNA probe cocktail (16, 18, 32, 34, 36, 39, 45, 51, 52, 56, 58, 59, 68). The light emitted from the conjugated hybrid antibody was measured in relative light units (RLU) using a luminometer. When the RLUs were equal to or greater than the mean of a positive control (RLU; 1.0 pg/mL), the sample was scored as positive.

Follow-up was carried out every 6 months for the first 2 years and every year for over 3 years, using cervical cytology and colposcopy. The HPV study was performed after 6 months. If the surgical margins were involved, the first control visit was scheduled for 3 months after treatment.

Patients with abnormal cytology results, suspicious colposcopy findings, or positive HPV results underwent a cervical biopsy. Patients with two consecutive negative Pap tests and a normal colposcopy were scheduled for annual gynecological visits.

### 2.3. Persistent/Recurrent Disease Criteria

Recurrence was defined as CIN2–3, determined by an exocervical biopsy or endocervical curettage. Patients with residual/recurrent disease underwent a second surgical treatment.

### 2.4. Cone Volume

The cone volume was estimated according to the formula proposed by Carcopino [8]. The circumference corresponded to the external circumference of the cone, the length (represented by the distance from the external/distal margin to the internal/proximal margin of the open sample), and the thickness (defined as the distance from the stromal margin to the surface of the epithelium). The volume of the cone was calculated using the hemiellipsoid formula:

Volume = (1/2) × (4/3) × π × length × (circumference/2π) × thickness

### 2.5. Data Analysis

A case form was developed in ACCESS, and the data were entered prospectively. The variables were analyzed according to their types and summarized in tables. The nominal categorical variables are described in terms of the absolute frequency of cases, and the percentage distribution is described with respect to the total by category and the number of missing data. Continuous variables with normal distributions are described in terms of the number of cases, the mean, the standard deviation, and the number of missing data.

Continuous variables that did not present a normal distribution are described in terms of the number of cases, the median, the first and third quartiles, and the number of missing data.

To compare the percentages between patients, the following statistical tests were used: for continuous variables with a normal distribution, Student’s t-test; for continuous variables that did not follow a normal distribution, the Kruskal–Wallis test; and, for categorical variables, Fisher’s exact test.

The time to recurrence was assessed using the Kaplan–Meier method. The log-rank test was used to compare the hazard ratios between the study groups. The Cox proportional hazards model was used to determine the risk variables. Incidences and 95% confidence intervals (CIs) were calculated.

With the patient as the unit of analysis, the probability of recurrence was estimated, taking into account CIN2–3 and CIN1. The date of the first conization and the end date at the time of the last follow-up were taken as the baseline moments when determining the recurrence of CIN2–3 or CIN1. Using a Cox regression model, the cumulative complication incidence function was calculated, taking into account CIN2–3 and CIN1.

The role of CIN2–3 persistence as a competing risk for CIN2–3 recurrence was studied using the Fine and Gray models. The statistical program R (version 3.6.1 for Windows) was used to process and analyze the data.

## 3. Results

The mean age at diagnosis in the type 1 excision group was 37.1 ± 10.9 years; the mean age in the type 2 excision group was 35.4 ± 9.6 years; and the mean age in the type 3 group was 41.1 ± 10.8 years, with *p* = 0.017. A total of 54.6% of patients (124/227) had clear margins in the LLETZ sample, and 31.3% of patients (71/227) presented involved surgical margins. In 13.2% of patients (30/227), the resection margins were uncertain. Of the total group of women with involved margins, 32.7% (35/107) were included in the type 1 group, 35.1% (26/74) in the type 2 group, and 21.7% (10/46) in the type 3 group; this difference was found to be significant (*p =* 0.008). According to the Carcopino formula, the median volume was 3.72 cc for the whole group, 2.49 cc in the group subjected to the type 1 technique, 5.02 cc in the group subjected to the type 2 technique, and 8.90 cc in the group subjected to the type 3 technique; these differences were significant (*p* < 0.001). The median total circumference for the type 1 excision technique was 13.0 cm; for type 2 treatment, it was 12.5 cm; and, for type 3 treatment, it was 11.9 (*p* = 0.68). The length of the surgical specimen for the type 1 technique was 6.3 mm; for the type 2 technique, it was 11.3 mm; and, for the type 3 technique, it was 21.4 mm. Again, these differences were statistically significant (*p* < 0.001). A second excision (endocervical canal widening) was performed in 89.1% (41/46) of the women who underwent type 3 excision (Table 1).

The pre-LLETZ HR-HPV (Table 2) result was positive in 88.2% of the patients (141/160) across the whole group. There were no significant differences in the proportion of positive or negative test results among the three techniques (*p =* 0.699). The result of the first post-LLETZ HR-HPV test was positive in 22.4% of the patients (38/170) in the whole group and negative in 77.6% (132/170). In the type 1 excision group, the HR-HPV post-LLETZ test result was positive in 28.6% of the patients (22/77); for the type 2 technique, it was positive in 20.6% of the patients (12/58); and, in the type 3 excision group, it was positive in 11.4% of the patients (4/35). There were no significant differences among the groups (*p* = 0.24).

In the present study, the persistence/recurrence of CIN2–3 was observed in 12 cases (5.28%). The cure rate was 94.72% with the diathermic loop therapeutic procedure, and this rate reached 99.17% in completely excised cases with clear margins. Recurrence was significantly associated with the margin status: there were four recurrences in patients with involved margins (HR 7.60; 95% CI 0.84 to 68.4) and six recurrences in patients with uncertain margins (HR 35.3; CI 95% 4.14 to 301). The women without recurrence had a median volume of 3.71 cc, while in patients with recurrence, the median volume was 5.16 cc; this was not a significant difference (*p =* 0.204). The other dimensions—namely, the circumference, thickness, length, and second excision—were not associated with the risk of the persistence/recurrence of CIN2–3 (*p* > 0.05; Table 3).

The overall mean age of the patients was 37.4 years. The mean age among the women without recurrence was 37.0 years, and it was 43.9 years in the group with CIN2–3 recurrence (*p* = 0.064). The result of the first HR-HPV test in the non-recurrence group was negative in 80.8% (131/162) of the women and positive in 19.2% (31/162), while in the recurrence group, the HR-HPV test result was negative in 12.5% (1/8) of the patients and positive in 87.5% (7/8); this was a significant difference (HR 27.18; 95% CI 3.41–227). The result of the first post-LLETZ RLU HPV test in the non-recurrence group was negative in 82.6% (124/150), it was positive within the range of 1 to 100 RLU in 14.0% of the patients (21/150), and positive with RLU > 100 in 3.3% (5/150) of the patients. In the recurrence group, the HR-HPV RLU result was negative in 12.5% of the patients (1/8), positive within the range of 1 to 100 RLU in 25.0% (2/8), and positive with RLU > 100 in 62.5.0% of the patients (5/8), displaying statistical significance (HR 204; 95% CI 21.5–1940). In the type 1 excision group, recurrence was observed in 6.54% of the cases (7/107); in the type 2 excision group, recurrence was observed in 5.41% of cases (4/74); and, in the type 3 excision group, recurrence was observed in 2.17% of cases (1/46). These differences among the treatments did not reach statistical significance (*p* = 0.72; Table 4).

The median LLETZ volume was 4.43 cc in the patients with negative HR-HPV results and 3.20 cc in the patients with a positive result. The circumference was 12.4 cm in the women with negative HR-HPV results and 13.6 cm in the patients with positive results; these differences were not significant. A positive post-LLETZ HR-HPV result was only statistically associated with the thickness (*p* = 0.038). In the type 1 excision group, there was a 28.5% rate (22/77) of positive tests; in the type 2 excision group, this rate was 20.6% (12/58); and, in the type 3 excision group, it was 11.4% (4/35). These differences did not reach statistical significance (*p* = 0.24; Table 5).

**Figure 2:**
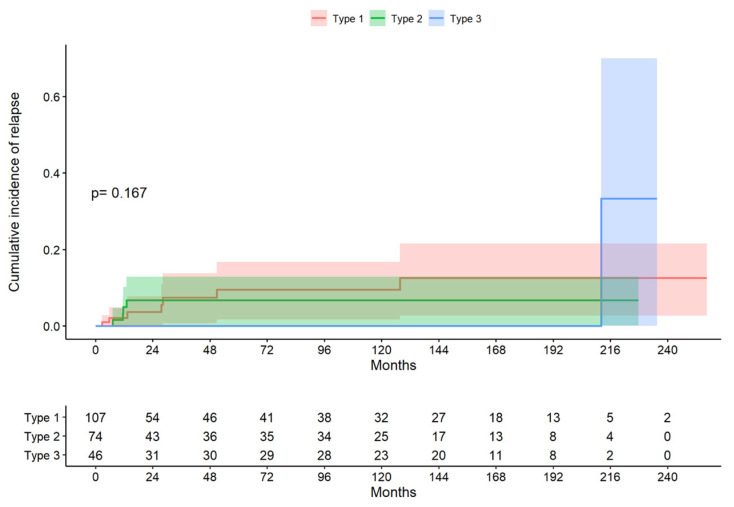
Cumulative incidence of relapse by excision type (descriptive figure presenting one of the data sets chosen at random).

The median follow-up period for the total group was 38.4 months (range 9.17 to 150 months). In the type 1 excision group, the median follow-up period (Q1; Q3) was 26.4 (7.69; 144) months; in the type 2 excision group, the median follow-up period (Q1; Q3) was 43.1 (12.1; 139) months; and, in the type 3 excision group, the median follow-up period (Q1; Q3) was 121 (11.8; 166) months. The log-rank test indicated that there was no evidence to reject the claim that the survival curves differed according to the treatment type (*p* = 0.167; Figure 2). The median time to recurrence in the whole group was 13.2 months (10.4; 33.9); in the type 1 treatment group, it was 27.7 months (9.54; 39.6); in the type 2 treatment group, it was 11.5 months (0.4; 12.0); and, in the type 3 treatment group, it was 212 months (212; 212). The probability of not presenting a recurrence at six months was 93.5%, the probability of presenting a CIN1 recurrence (LSIL) was 5.6%, and that of presenting a CIN2–3 recurrence (HSIL) was 0.9%.

## 4. Discussion

The most important findings were that the persistent/recurrent CIN2–3 was found in 12 cases (5.2%). Type 1 excision was performed in 107 patients, with 7 recurrences (6.5%); type 2 excision was performed in 74 patients, with 4 recurrences (5.4%); and type 3 excision was performed in 46 patients, with 1 recurrence (2.1%). The volume and dimensions of the cone were not associated with recurrence. Type 3 excision was associated with a larger proportion of clear margins and lower post-cone HR-HPV positivity, with a lower incidence of the persistence/recurrence of CIN2–3.

In this single-center retrospective study, we found that the age of patients was higher for type 3 excision (median 41.1 years) in relation to types 1 and 2, which corresponded to cases of type 3 TZ, which occurs due to the change in the visibility of the TZ from visible to non-visible in women over 40 years, as reported by Desai [9]. We assessed the status of the margins in 227 women according to the type of resection. The endocervical margins were clear in 86.8% of the patients; in the type 1 excision group, 9.35% (10/107) had endocervical margin involvement; in the type 2 excision group, this rate was 14.9% (11/74); and, in the type 3 excision group, it was 2.17% (1/46). Extensive involvement of the endocervical margin after LLETZ in cases of CIN2–3 is a strong predictor of residual disease [10]. Arbyn [1] reported a risk of persistence/recurrence of 7.2% if only the exocervical margin was involved and 16.3% if the endocervical margin was involved; however, if both were involved, the recurrence rate was 18.9% [1]. We found a risk of persistent recurrence of 0.81% if the margins were clear, 2.4% if only the exocervical margin was affected, 9% if the endocervical margin was involved, and 12.5% if both the exocervical and endocervical margins were involved. Therefore, recurrence is greater if both margins are involved. In addition, the median time to recurrence in the patients with clear margins was 128 months; that in women with exocervical and endocervical margin involvement was 12.3 months; and, in those with uncertain margins, it was 20.5 months. In summary, the patients who had both margins involved had a greater probability of persistent recurrence, and it was likely to occur in a shorter time.

In the type 1 excision group, 32.7% (35/107) had involved global (endocervical or exocervical) margins; in the type 2 excision group, this rate was 35.1% (26/74); and, in the type 3 excision group, it was 21.70% (10/46). However, the persistence/recurrence rate was 6.54% (7/107) in the type 1 excision group, 5.41% (4/74) in the type 2 excision group, and 2.17% (1/46) in the type 3 excision group. Not all patients with involved margins presented persistence/recurrence, but the type 3 group presented a lower proportion of involved margins and less persistence/recurrence as, in this technique, the surgical piece had a greater depth (2.14 cm) and volume, which could optimally eliminate HPV. Among those with clear margins, recurrence occurred in 0.81% (one case) in 128 months, but if involved endocervical and exocervical margins were present, 5% (four cases) experienced recurrences within a median time period of 12.3 months. In those with uncertain margins, 20.0% (six cases) experienced recurrences in 20.5 months. Alder [11] demonstrated that women with involved/uncertain margins had a higher risk of recurrent CIN2–3, which was worse than that in women with negative margins, and Lubrano [12] reported persistence/recurrence in 24.8% of cases with involved margins vs. 11.1% of cases with negative margins (*p* < 0.0001). Feng [13] reported that the residual/recurrence rate was significantly higher in patients with involved endocervical margins than in patients with negative endocervical margins (OR = 2.59, *p* < 0.00001), which could be explained by the persistence of high-risk human papillomavirus infections in the endocervical glands [13]. Giannini [14] demonstrated, through a multivariable analysis, that only involved endocervical rather than ectocervical margins (HR: 4.56; 95% CI: 1.23–7.95; *p* = 0.021) were associated with worse outcome.

The median circumference and the thickness were similar between the three types of excision, but the volume and length (depth) of the surgical specimen were greater in the type 3 excision group than in the other two groups, which was related to the lower percentage of margin involvement for type 3 excision. Papoutsis [15] determined that, for prediction of the involved margins, the optimal cone volume was 2.1 cc and the optimal cone length (depth) was 1.0 cm. In our study, the volume of the LLETZ in the type 1 treatment group was 2.49 cc, while that in the type 2 excision group was 5.02 and that in the type 3 excision group was 8.9 cc. This latter result was higher than the results obtained for the other two techniques, which indicated a higher percentage of clear margins (69.6%) in the type 3 excision group. When determining the state of the margins, the length (depth) is probably more important than the volume of the cone, as it allows for a clear endocervical margin and endocervical reserve cells free of human papillomavirus infection.

Kawano [16] estimated that the optimal cut-off point was 15 mm in the case of single-quadrant disease and 20 mm when two or more quadrants were involved. In a 2013 Korean retrospective cohort study of 1220 women—289 women (24%) with CIN2, 916 women (75%) with CIN3, and 15 women (1%) with stage 1a1 cervical cancer—Bae [17] suggested that the cone length should be adjusted according to the patient’s age and grade of the lesion (<40 years and CIN2: ≥9 mm; 40–50 years and CIN2: ≥12 mm; <50 years and CIN3 or stage 1a1 cervical cancer: ≥18 mm). A cone length of at least 18 mm was required to achieve the resection of a clear endocervical margin in 86% of women younger than 50 years with CIN2+ and in 88% of women younger than 40 years with CIN2+. For CIN2, a 9 mm cone length achieved clearance in 83% of women younger than 50 years, whereas a cone length of 12 mm was required to achieve clearance in 90% of women younger than 50 years [17]. Although recommendations have been published on the optimal length of the LLETZ according to the type of ZT, there is no consensus, the reported measurements vary between 9 mm and 20 mm, and the ideal length should be evaluated taking into account the type of transformation zone [18].

The post-LLETZ HR-HPV result was negative in 88.5% (31/35) of cases in the type 3 excision group, which was a higher rate than in the type 1 (71.4%) and type 2 (79.3%) excision groups. The type 3 group also had the lowest percentage of post-LLETZ HR-HPV positivity of 11.4% (4/35), which could be explained by the greater volume and length of the surgical specimens removed through type 3 excision, with a median length of 21.4 mm. The removal of a greater amount of endocervical tissue could lead to resection of the disease in its entirety and successfully eliminate the virus from the cervical tissue. Meanwhile, in type 1 and type 2 excision—which are performed at shallower depths—it is likely that not all of the HPV-infected tissue is removed. Therefore, both groups presented a higher viral load in the RLU HPV test at 6 months and post-LLETZ HR-HPV positivity. Endocervical gland involvement in histological samples is associated with increased rates of HR-HPV infections and CIN2+ lesions and also showed increased CIN recurrence/persistence after treatment compared to controls [19]. Among women who underwent a cervical biopsy, a higher HR-HPV viral load in the cervical lesions was associated with a higher risk of high-grade cervical lesions [20]. Excision of HPV-infected proliferating endocervical reserve cells, which may have oncogenic potential, could decrease the possibility of CIN2–3 recurrence [21].

Persistence/recurrence was associated with the status of the surgical margins. In our study, we found a risk of 0.81% if the margins were clear, 5.63% if the margins were involved, and a 20% risk of recurrence if the margins were uncertain (*p* < 0.001). Similarly, Alder [11] reported a significant association between persistence/recurrence and involved/uncertain margins. Persistence/recurrence was not associated with the volume or dimensions of the surgical specimen, as indicated in the results described by Chen [22], who found no association with the circumference, thickness, or length of the surgical piece. Rather, it was found that positive margins—particularly, endocervical margins—as well as abnormal cytology and a positive HR-HPV test during follow-up were risk factors for persistent HSIL lesions after LEEP conization. Ultimately, the most important factor is the state of the margin, especially the endocervical margin, regardless of the other dimensions. The persistence/recurrence group had a greater volume than the non-recurrence group but, despite the removal of a greater amount of tissue, the patients had a greater proportion of persistence/recurrence, which could be explained by the more extensive lesions and more aggressive HPV genotypes, as well as the fact that the surgeon, upon colposcopically observing a larger lesion, performed larger-volume LLETZ. Peng [23] reported that a positive margin (*p* < 0.001) and multiple quadrant involvement (*p* < 0.001) were identified as independent risk factors for residual lesions in a multivariate analysis. Fernández [24] reported that cases positive for HR-HPV recur sooner, as do those with compromised margins; however, HR-HPV appears to be the most powerful predictor of treatment failure.

In our study, it was determined that a positive result on the first post-LLETZ HR-HPV test was associated with the risk of persistence/recurrence, with statistical significance (*p* < 0.001). These data coincide with those of Arbyn [1], who demonstrated that the results of post-LLETZ HR-HPV testing predict treatment failure more accurately than the margin status. Huang [25] observed a greater recurrence of CIN2+ in patients with positive results for post-LLETZ HR-HPV, reaching 14.8% at 5 years, and Bogani [26] found that the persistence of HPV positivity after LLETZ was the main predictive factor of CIN2+, regardless of the HPV genotype. In our study, patients with a viral load >100 URL/mL post-LLETZ had a 204 times higher risk of recurrence, when compared to women with a viral load <100 URL/mL. However, in the study by Fu [27], taking the average age and HR-HPV viral load tested via hybrid Capture 2 (HC2) as thresholds, the risk of the persistence/recurrence of CIN2+ was higher in women aged over 40 years or with a baseline HC2 of 300 RLU or more. The detection of a viral infection may also be affected by the detection method, the sampling site, and the number of epithelial cells in the sample. The persistence/recurrence rate was 6.54% (7/107) in the type 1 excision group, 5.41% (4/74) in the type 2 excision group, and 2.17% (1/46) in the type 3 excision group as, in TZ type 3, the surgical specimen had a greater volume and length; however, this difference was not statistically significant.

The volume, circumference, and length of the LLETZ specimen were not associated with post-LLETZ HR-HPV positivity; the only significant association was found with the thickness, perhaps as patients with more extensive lesions had a higher viral load and more aggressive genotypes associated with a high risk. The type 3 excision group (11.4%) had the lowest percentage of HR-HPV positivity post-LLETZ, compared to the type 1 (28.5%) and type 2 (20.6%) excision groups, perhaps as a clear endocervical margin was achieved through obtaining a longer surgical specimen. Ding [28] found that women with involved margins had a more severe pathology at the baseline diagnosis (*p* < 0.001) and had a higher risk of HR-HPV infection after LLETZ (*p* < 0.001). The median time to recurrence in the whole group was 13.2 months. Kulkarni [29] reported an overall persistence/recurrence rate of 11.3%, where the median time to persistence/recurrence was 6.5 months, and multivariate regression models demonstrated that follow-up HPV positivity (OR = 22.0) and positive margins (OR = 3.7) were significantly associated with persistence/recurrence. Kocken [30] reported that the 5-year risk of CIN grade 2 or higher after treatment in women with three consecutive negative cytology tests or negative co-tests for cytology and hrHPV at 6 and 24 months was similar to that of women with normal cytology at 6 and 24 months.

The major strength of this study is the large number of cases analyzed, which provides an overview of the data from a tertiary referral center; however, the retrospective analysis, long study period, missing cases, and low incidence of recurrence can be considered limitations that may affect the results.

## 5. Conclusions

According to the obtained results, type 3 excision was associated with a larger proportion of clear margins and a smaller proportion of positivity for HR-HPV post-LLETZ, with a lower incidence of the persistence/recurrence of CIN2–3.

## Figures and Tables

**Table 1:** Characteristics of patients and surgical specimens according to the type of treatment and follow-up.

	[All]	Type 1	Type 2	Type 3	*p*. Overall	N
	N = 227	N = 107	N = 74	N = 46		
**Patient characteristics**						
Age (years), mean (standard deviation)	37.4 (10.7)	37.1 (10.9)	35.4 (9.68)	41.1 (10.8)	0.017	227
**Surgical specimen characteristics**						
Margin status, N (%):					0.016	227
Clear	124 (54.6%)	48 (44.9%)	44 (59.5%)	32 (69.6%)		
Ectocervical+	41 (18.1%)	20 (18.7%)	14 (18.9%)	7 (15.2%)		
Endocervical+	22 (9.69%)	10 (9.35%)	11 (14.9%)	1 (2.17%)		
All margins	8 (3.52%)	5 (4.67%)	1 (1.35%)	2 (4.34%)		
Uncertain	30 (13.2%)	22 (20.6%)	4 (5.41%)	4 (8.70%)		
Unknown	2 (0.88%)	2 (1.87%)	0 (0.00%)	0 (0.00%)		
Margins categorized, N (%):					0.008	227
Clear	124 (54.6%)	48 (44.9%)	44 (59.5%)	32 (69.6%)		
* Involved	71 (31.3%)	35 (32.7%)	26 (35.1%)	10 (21.70%)		
Uncertain	30 (13.2%)	22 (20.6%)	4 (5.41%)	4 (8.70%)		
Unknown	2 (0.88%)	2 (1.87%)	0 (0.00%)	0 (0.00%)		
Median LLETZ volume (Q1; Q3) cc	3.72 (2.20; 6.48)	2.49 (1.51; 4.29)	5.02 (3.31; 6.16)	8.90 (7.94; 11.1)	0.001	105
Median circumference (Q1; Q3) cm	11.1 (9.16; 13.7)	11.5 (8.63; 16.0)	10.8 (9.59; 12.3)	11.2 (9.16; 12.6)	0.99	129
Thickness (Q1; Q3) cm	1.05 (0.92; 1.25)	1.05 (0.90; 1.25)	1.05 (1.00; 1.25)	1.10 (0.96; 1.30)	0.473	227
Length, mean (standard deviation) cm	1.02 (0.55)	0.63 (0.18)	1.13 (0.15)	2.14 (0.35)	<0.001	105
Second excision, N (%)	68 (30.0%)	12 (11.2%)	15 (20.3%)	41 (89.1%)	<0.001	227
Follow-up (months), media (Q1; Q3)	38.4 (9.17; 150)	26.4 (7.69; 144)	43.1 (12.1; 139)	121 (11.8; 166)	0.088	227
Time to relapse (months), media [Q1; Q3]	13.2 (10.4; 33.9)	27.7 (9.54; 39.6)	11.5 (0.4; 12.0)	212 (212; 212)		12

* HR-HPV = high-risk human papillomavirus, Involved = endocervical and exocervical and positive margins, LLETZ = large-loop excision of the transformation zone.

**Table 2:** High-risk human papillomavirus (HR-HPV) results, according to type of excision.

	[ALL]	Type 1	Type 2	Type 3	*p*. Overall	N
	N = 227	N = 107	N = 74	N = 46		
* HPV Results						
Previous HPV, N (%):					0.699	227
Negative	19 (11.8%)	8 (10.9%)	6 (10.7%)	5 (16.1%)		
Positive	141 (88.2%)	65 (89.1%)	50 (89.2%)	26 (83.9%)		
Unknown	67	34	18	15		
First post-LLETZ HR-HPV test, N (%):					0.24	226
Negative	132 (77.6%)	55 (71.4%)	46 (79.3%)	31 (88.5%)		
Positive	38 (22.4%)	22 (28.5%)	12 (20.6%)	4 (11.4%)		
Unrealized	56	30	15	11		
First post-LLETZ RLU HPV test cat, N (%):					0.315	227
Negative	125 (79.1%)	51 (71.8%)	45 (83.3%)	29 (87.8%)		
1–100	23 (14.5%)	14 (19.7%)	5 (9.2%)	4 (12.1%)		
>100	10 (6.3%)	6 (8.4%)	4 (7.4%)	0 (0.00%)		
Unknown	69	36	20	13		

* HPV = human papillomavirus, LLETZ = large-loop excision of the transformation zone, RLU = relative light units.

**Table 3:** Persistence/recurrence according to the characteristics of the surgical specimens from patients with cervical intraepithelial neoplasm 2–3, treated using the large-loop excision of the transformation zone (LLETZ).

	[Total]	No Persistence/Recurrence	Persistence/Recurrence	HR	*p*. Overall	N
	N = 227	N = 215	N = 12			
Margin status N (%):					<0.001	242
Clear	124 (54.6%)	123 (99.2%)	1 (0.81%)	Ref.		
* Involved	71 (31.0%)	67 (94.4%)	4 (5.63%)	7.60 [0.84; 68.4]	0.07	
Ectocervical+	41 (18.1%)	40 (97.6%)	1 (2.4%)		0.43	
Endocervical+	22 (9.69%)	20 (90.9%)	2 (9.1%)		0.12	
Both margins+	8 (3.52%)	7 (87.5%)	1 (12.5%)		0.059	
Uncertain	30 (13.2)	24 (80.0%)	6 (20.0%)	35.3 [4.14; 301]	0.001	
Unknown	2 (0.88%)	1 (50.0%)	1 (50.0%)	88.4 [5.40; 1448]	0.002	
Median LLETZ volume (Q1; Q3)	3.72 (2.20; 6.48)	3.71 (2.18; 6.40)	5.16 (4.24; 10.2)	1.13 [0.94; 1.36]	0.204	105
Circumference, mean (standard deviation) cm	9.6 (5.61)	9.5 (5.39)	13.0 (11.2)	1.07 [0.95; 1.21]	0.262	129
Thickness, mean (standard deviation) cm	1.13 (0.35)	1.12 (0.36)	1.25 (0.25)	1.36 [0.39; 4.72]	0.625	227
Length, median (standard deviation) cm	1.02 (0.55)	1.02 (0.56)	0.97 (0.15)	0.92 [0.07; 12.3]	0.951	105
Second excision						
N (%)	159 (70.0%)	151 (95.0%)	8 (5.03%)	Ref.	Ref.	
N (%)	68 (30.0%)	64 (94.1%)	4 (5.88%)	1.11 [0.33; 3.69]	0.871	227

* Involved exocervical, endocervical, and both positive margins, LLETZ = large-loop excision of transformation zone.

**Table 4:** Relationship between post-LLETZ HR-HPV result and type of excision and persistence/recurrence of cervical intraepithelial neoplasm (CIN2–3) in patients affected by CIN2–3 and treated with large-loop excision of transformation zone (LLETZ).

	[Total]	No Persistence	Persistence	HR	*p*. Overall	N
		Recurrence	Recurrence			
	N = 227	N = 215	N = 12			
* First post-LLETZ HR-HPV, N (%):						226
Negative	132 (77.6%)	131 (80.8%)	1(12.5%)	Ref.	Ref.	
Positive	38 (22.3%)	31 (19.2%)	7 (87.5%)	27.8 [3.41; 227]	0.002	
Unrealized	56	52	4	6.52 [0.70; 61.1]	0.101	
First post-LLETZ RLU HPV categories, N (%):						227
Negative	125 (79.1%)	124 (82.6%)	1 (12.5%)	Ref.	Ref.	
1–100	23 (14.5%)	21 (14.0%)	2 (25.0%)	10.3 [0.93; 114]	0.058	
>100	10 (6.3%)	5 (3.3%)	5 (62.5%)	204 [21.5; 1940]	<0.001	
Unrealized	69	65	4	4.60 [0.49; 43.5]	0.182	
**Type of excision**						227
**N** (**%**):						
Type 1	107 (47.1%)	100 (93.5%)	7 (6.54%)	Ref.	Ref.	
Type 2	74 (32.6%)	70 (94.6%)	4 (5.41%)	0.94 [0.15; 5.86]	0.94	
Type 3	46 (20.3%)	45 (97.8%)	1 (2.17%)	0.64 [0.03; 15.7]	0.723	

* HPV = human papillomavirus, LLETZ = large-loop excision of the transformation zone.

**Table 5:** Relationship between the volume, dimensions, and type of excision and post-LLETZ HR-HPV positivity in patients affected by cervical intraepithelial neoplasm (CIN2–3) and treated with the large-loop excision of the transformation zone (LLETZ).

HPV	[All]	Negative	Positive	Unrealized	*p*. Overall	N
	N = 226	N = 132	N = 38	N = 56		
Median LLETZ volume (Q1; Q3)	3.71 (2.19; 6.54)	4.43 (2.10; 7.28)	3.20 (2.39; 4.56)	3.62 (2.48; 4.99)	0.607	104
Circumference, mean (standard deviation) cm	12.6 (5.63)	12.4 (5.02)	13.6 (8.50)	12.5 (4.59)	0.709	128
Thickness, mean (standard deviation) cm	1.13 (0.35)	1.09 (0.28)	1.10 (0.32)	1.23 (0.48)	0.038	226
Length, mean (standard deviation) cm	1.02 (0.55)	1.08 (0.61)	0.85 (0.28)	0.79 (0.20)	0.135	104
Type of excision, N (%):					0.24	226
Type 1	107 (47.3%)	55 (71.4%)	22 (28.5)	30 (53.6)		
Type 2	73 (32.3%)	46 (79.3%)	12 (20.6)	15 (26.8)		
Type 3	46 (20.4%)	31 (88.5%)	4 (11.4)	11 (19.6)		

CIN = cervical intraepithelial neoplasm, LLETZ = large-loop excision of the transformation zone.

## Data Availability

The data presented in this study are available on request from the corresponding author.

## References

[1] Arbyn M., Redman C.W., Verdoodt F., Kyrgiou M., Tzafetas M., Ghaem-Maghami S., Petry K.-U., Leeson S., Bergeron C., Nieminen P. (2017). Incomplete excision of cervical precancer as a predictor of treatment failure: A systematic review and meta-analysis. Lancet Oncol..

[2] Desravines N., Miele K., Carlson R., Chibwesha C., Rahangdale L. (2020). Topical therapies for the treatment of cervical intraepithelial neoplasia (CIN) 2–3: A narrative review. Gynecol. Oncol. Rep..

[3] Basu P., Taghavi K., Hu S.Y., Mogri S., Joshi S. (2018). Management of cervical premalignant lesions. Curr. Probl. Cancer.

[4] NHSCSP NHS Cervical Screening Program Colposcopy and Program Management NHSCSP Publication No. 20, 3rd Edition March 2016. *NHS Cerv. Screen Program*. 3rd Ed.; 2016, Volume 20, pp. 1–108. https://www.bsccp.org.uk/assets/file/uploads/resources/NHSCSP_20_Colposcopy_and_Programme_Management_(3rd_Edition)_(2).pdf.

[5] Torné Bladé A., AEPCC (2014). SEGO Oncoguide: Prevention of cervical cancer. Clinical practice guidelines in gynecological and breast cancer. Span. Assoc. Patol. Cerv. Colposc..

[6] Bornstein J., Bentley J., Bösze P., Girardi F., Haefner H., Menton M., Perrotta M., Prendiville W., Russell P., Sideri M. (2012). 2011 Colposcopic Terminology of the International Federation for Cervical Pathology and Colposcopy. Obstet. Gynecol..

[7] Solomon D., Davey D., Kurman R., Moriarty A., O’Connor D., Prey M., Raab S., Sherman M., Wilbur D., Wright J.T. (2002). The 2001 Bethesda System: Terminology for reporting results of cervical cytology. J. Am. Med. Assoc..

[8] Carcopino X., Mancini J., Prendiville W., Gondry J., Chevreau J., Lamblin G., Atallah A., Lavoue V., Caradec C., Baldauf J.-J. (2017). The Accuracy of Large Loop Excision of the Transformation Zone Specimen Dimensions in Determining Volume: A Multicentric Prospective Observational Study. J. Low. Genit. Tract. Dis..

[9] Desai K., Hansen N., Rodriguez A.C., Befano B., Egemen D., Gage J.C., Wentzensen N., Lopez C., Jeronimo J., San Jose S. (2024). Squamocolumnar junction visibility, age, and implications for cervical cancer screening programs. Prev. Med..

[10] Kietpeerakool C., Khunamornpong S., Srisomboon J., Siriaunkgul S., Suprasert P. (2007). Cervical intraepithelial neoplasia II-III with endocervical cone margin involvement after cervical loop conization: Is there any predictor for residual disease?. J. Obstet. Gynaecol. Res..

[11] Alder S., Megyessi D., Sundström K., Östensson E., Mints M., Belkić K., Andersson S. (2020). Incomplete excision of cervical intraepithelial neoplasia as a predictor of the risk of recurrent disease—A 16-year follow-up study. Am. J. Obstet. Gynecol..

[12] Lubrano A., Medina N., Benito V., Arencibia O., Falcón J.M., Leon L., Molina J., Falcón O. (2012). *Follow-up after LLETZ: A study of* 682 cases of CIN 2-CIN 3 in a single institution. Eur. J. Obstet. Gynecol. Reprod. Biol..

[13] Feng H., Chen H., Huang D., He S., Xue Z., Pan Z., Huang Y. (2022). Relationship between positive margin and residual/recurrence after excision of cervical intraepithelial neoplasia: A systematic review and meta-analysis. Transl. Cancer Res..

[14] Giannini A., Di Donato V., Sopracordevole F., Ciavattini A., Ghelardi A., Vizza E., Bogani G. (2023). Outcomes of High-Grade Cervical Dysplasia with Positive Margins and HPV Persistence after Cervical Conization. Vaccines.

[15] Papoutsis D., Rodolakis A., Mesogitis S., Sotiropoulou M., Antsaklis A. (2013). Appropriate cone dimensions to achieve negative excision margins after large loop excision of transformation zone in the uterine cervix for cervical intraepithelial neoplasia. Gynecol. Obstet. Investig..

[16] Kawano K., Tsuda N., Nishio S., Yonemoto K., Tasaki K., Tasaki R., Ushijima K. (2016). Identification of appropriate cone length to avoid positive cone margin in high grade cervical intraepithelial neoplasia. J. Gynecol. Oncol..

[17] Bae H.S., Chung Y.W., Kim T., Lee K.W., Song J.Y. (2023). The appropriate cone depth to avoid endocervical margin involvement is dependent on age and disease severity. Acta Obs. Gynecol. Scand..

[18] Kyrgiou M., Athanasiou A., Arbyn M., Lax S.F., Raspollini M.R., Nieminen P., Carcopino X., Bornstein J., Gultekin M., Paraskevaidis E. (2022). Terminology for cone dimensions after local conservative treatment for cervical intraepithelial neoplasia and early invasive cervical cancer: 2022 consensus recommendations from ESGO, EFC, IFCPC, and ESP. Lancet Oncol..

[19] Spinillo A., Dominoni M., Boschi A., Cesari S., Fiandrino G., Gardella B. (2020). The relationship of human papillomavirus infection with endocervical glandular involvement on cone specimens in women with cervical intraepithelial neoplasia. Gynecol. Oncol..

[20] Liu Y., Xu C., Pan J., Sun C., Zhou H., Meng Y. (2021). Significance of the viral load of high-risk HPV in the diagnosis and prediction of cervical lesions: A retrospective study. BMC Womens Health.

[21] Regauer S., Reich O., Kashofer K. (2022). Cervical Precancers Originate from Infected Proliferating Reserve Cells: A Comparative Histologic and Genetic Study of Thin and Thick High-grade Squamous Intraepithelial Lesions. Am. J. Surg. Pathol..

[22] Chen L., Liu L., Tao X., Guo L., Zhang H., Sui L. (2019). Risk Factor Analysis of Persistent High-Grade Squamous Intraepithelial Lesion after Loop Electrosurgical Excision Procedure Conization. J. Low. Genit. Tract. Dis..

[23] Peng H., Liu W., Jiang J., Du H. (2023). Extensive lesions and a positive cone margin are strong predictors of residual disease in subsequent hysterectomy following conization for squamous intraepithelial lesion grade 2 or 3 study design. BMC Womens Health.

[24] Fernández-Montolí M.E., Tous S., Medina G., Castellarnau M., García-Tejedor A., de Sanjosé S. (2020). Long-term predictors of residual or recurrent cervical intraepithelial neoplasia 2-3 after treatment with a large loop excision of the transformation zone: A retrospective study. BJOG Int. J. Obstet. Gynaecol..

[25] Huang H., Tung H., Yang L., Chao A., Tang Y., Chou H., Chang W., Wu R., Huang C., Lin C. (2021). Role of human papillomavirus status after conization for high-grade cervical intraepithelial neoplasia. Int. J. Cancer.

[26] Bogani G., Pinelli C., Chiappa V., Martinelli F., Lopez S., Ditto A., Raspagliesi F. (2020). Age-specific predictors of cervical dysplasia recurrence after primary conization: Analysis of 3212 women. J. Gynecol. Oncol..

[27] Fu K., Lei M., Yang W.Q., Wu L.S., Shi J.C., Zhang Y. (2022). The treatment strategy of patients with positive margins after cervical cold knife conization-A 7-year retrospective study in China. Int. J. Gynaecol. Obstet..

[28] Ding T., Li L., Duan R., Chen Y., Yang B., Xi M. (2023). Risk factors analysis of recurrent disease after treatment with a loop electrosurgical excision procedure for high-grade cervical intraepithelial neoplasia. Int. J. Gynaecol. Obstet..

[29] Kulkarni A., Covens A., Durand N., Ghorab Z., Gien L.T., Osborne R., Vicus D., Kupets R. (2023). Role of HPV in the Prediction of Persistence/Recurrence After Treatment for Cervical Precancer. J. Obs. Gynaecol. Can..

[30] Kocken M., Helmerhorst T.J., Berkhof J., Louwers J.A., Nobbenhuis M.A., Bais A.G., Hogewoning C.J., Zaal A., Verheijen R.H., Snijders P.J. (2011). Risk of recurrent high-grade cervical intraepithelial neoplasia after successful treatment: A long-term multi-cohort study. Lacet Oncol..

